# Current Trends and Technological Advancements in the Use of Oxalate-Degrading Bacteria as Starters in Fermented Foods—A Review

**DOI:** 10.3390/life14101338

**Published:** 2024-10-21

**Authors:** Sajad Hamid Al-Kabe, Alaa Kareem Niamah

**Affiliations:** Department of Food Science, College of Agriculture, University of Basrah, Basra City 61004, Iraq; pgs.sajad.hamed@uobasrah.edu.iq

**Keywords:** probiotic bacteria, gut bacteria, kidney stone, oxalate metabolism

## Abstract

Nephrolithiasis is a medical condition characterized by the existence or development of calculi, commonly referred to as stones within the renal system, and poses significant health challenges. Calcium phosphate and calcium oxalate are the predominant constituents of renal calculi and are introduced into the human body primarily via dietary sources. The presence of oxalates can become particularly problematic when the delicate balance of the normal flora residing within the gastrointestinal tract is disrupted. Within the human gut, species of *Oxalobacter*, *Lactobacillus*, and *Bifidobacterium* coexist in a symbiotic relationship. They play a pivotal role in mitigating the risk of stone formation by modulating certain biochemical pathways and producing specific enzymes that can facilitate the breakdown and degradation of oxalate salts. The probiotic potential exhibited by these bacteria is noteworthy, as it underscores their possible utility in the prevention of nephrolithiasis. Investigating the mechanisms by which these beneficial microorganisms exert their effects could lead to novel therapeutic strategies aimed at reducing the incidence of kidney stones. The implications of utilizing probiotics as a preventive measure against kidney stone formation represent an intriguing frontier in both nephrology and microbiome research, meriting further investigation to unlock their full potential.

## 1. Introduction

The condition known as nephrolithiasis is characterized by solid deposits in the urinary system, commonly called stones, which hinder normal urine flow. These deposits form due to elevated levels of calcium, oxalic acid, phosphate, urate, and cystine in the urine. Regrettably, the prevalence of urolithiasis has significantly risen in the last three decades, possibly attributed to environmental shifts such as suboptimal dietary habits and reduced physical exertion. Presently, nephrolithiasis holds the third position among the most prevalent urological disorders. Urolithiasis varies from 7 to 13% in North America, 5 to 9% in Europe, and 1 to 5% in Asia. Nephrolithiasis primarily affects males aged 40 to 50 and females aged 50 to 70 [1]. Deposits found in the urinary system exhibit variability in their chemical makeup; the majority of stones, approximately 80%, consist of calcium, while 9% are composed of uric acid, 10% of struvite, and 1% of cysteine. Within the category of calcium stones, half are calcium oxalate, 5% are calcium phosphate, and the remaining 45% are a combination of both types. The process of kidney stone formation is intricate and influenced by various factors, including intrinsic ones such as age, gender, and genetic predisposition, as well as extrinsic factors like geographical location, climate, dietary habits, mineral content, and water consumption patterns [2]. Oxalic acid (pKa = 1.2), a potent organic acid, is abundantly present in nature and can be found in various plants and animals. Within plants, it typically builds up as a metabolic byproduct in the shape of free acid, serving various functions within the organism. Interestingly, oxalic acid also exhibits chelating properties towards cations and is frequently encountered in the form of soluble sodium or potassium oxalate. Moreover, it tends to precipitate as insoluble calcium oxalate, further showcasing its diverse chemical behavior in biological systems [3,4].

The primary objective of the present review was to categorize the various biological techniques that can potentially decrease the levels of oxalates found in food products while also examining the methodologies employed for the quantification of oxalates in these food items. We have performed a literature search for oxalate-degrading bacteria using the internet and authenticated research articles, especially focusing on the role of lactic acid bacteria and bifidobacteria. We also tried to analyze the major outcomes of the different studies and compile them in tabular form for ease of understanding and discuss them comprehensively in the text.

Oxalic acid and its oxalate salts are present in the blood (plasma) and urine of both animals and humans [1,4]. It is noteworthy that a portion of the oxalate observed in the human body is obtained via the consumption of oxalate-rich plant material, primarily through the intake of strawberries, spinach, rhubarb, beets, nuts, wheat bran, chocolate, tea, and coffee, as outlined in Table 1. The concentration of oxalate in plant foods can vary for several reasons, such as cultivar, harvesting time, processing, etc. It is also reported that free calcium can reduce soluble oxalate in food by forming insoluble oxalate [5].

It has been observed that the processing method can significantly impact oxalate levels in different plant-derived foods. For instance, boiling plant leaves can leach out soluble oxalates; hence, it reduces oxalate levels. Similarly, hot-air drying can also reduce oxalate levels in rice paddy herbs [5]. Oxalate-degrading microorganisms also reduce their levels via biochemical reactions mediated by the production of enzymes, and, therefore, fermentation may also observe a similar effect.

**Figure 1:**
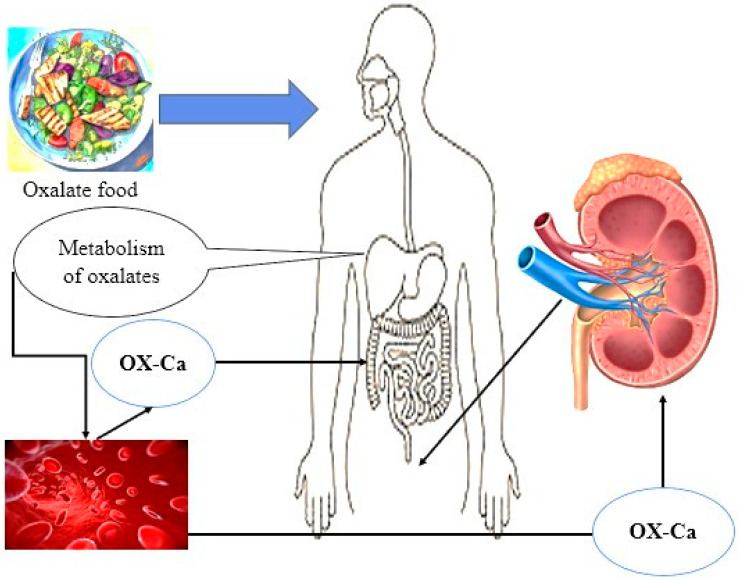
Food and internal sources (liver metabolism) are the two main sources of oxalate in the body.

Simultaneously, a certain quantity of oxalate is internally generated in the liver as a byproduct of the metabolic processes involving glycine, glyoxylate, and ascorbic acid [6]. Despite this understanding, the precise ratios at which exogenous and endogenous origins contribute to the overall oxalate levels in the gastrointestinal tract remain a topic of ongoing discussion and investigation (Figure 1). This debate underscores the complexity of oxalate metabolism and the importance of understanding the sources and pathways through which oxalate is introduced into the human system, thereby emphasizing the need for further research in this area [7].

The intricate interplay between dietary intake, internal production, and physiological mechanisms governing oxalate levels represents the multifaceted nature of oxalate homeostasis and the necessity for a holistic approach to exploring the factors influencing oxalate concentrations in the body [8]. In light of the potential implications for health and disease, elucidating the nuanced dynamics of oxalate sources and their impact on bodily processes is crucial for advancing our knowledge in this field and informing strategies for managing oxalate-related conditions. *Oxalobacter formigenes* is recognized as a bacterium that degrades oxalate, utilizing intestinal oxalate as its exclusive source of carbon to regulate oxalate homeostasis within the biological system. Nevertheless, the utilization of this bacterium as a probiotic has been constrained primarily due to its demanding nutritional requirements, limited capacity for colonization, and highly specialized oxalotrophic characteristics [9]. Conversely, lactic acid bacteria (LAB) constitute essential components of the human intestinal environment and have been widely employed as probiotics owing to their advantageous effects on the health of the host organism [10,11]. Various research studies have validated the association between the oral intake of *Lactobacillus* or *Bifidobacterium* species and their significant involvement in reducing luminal oxalate levels, consequently leading to a decline in the probability of urinary oxalate excretion in both human subjects and animals [12,13]. Furthermore, this review aimed to compile and showcase the diverse strategies utilized by different researchers across the world for the reduction of oxalates and to ascertain the different approaches for determining the presence of oxalates in food samples.

**Table 1:** Percentage of oxalate concentration in some types of food and beverages.

Food and Beverage Types	Concentration of Oxalate (mg/100 g or mL)	References
*Citrus aurantium*	2.07–10.64	[14]
*Musa acuminata* *Musa balbisiana*	0.00–9.90	
*Spinacia oleracea*	364.44–1145.00	
*Beta vulgaris*	36.90–794.12	
*Arachis hypogaea*	64.57–348.58	
*Glycyrrhiza glabra*	3343.20–3795.40	[15]
*Phaseolus vulgaris*	500.60–593.20	
*Oryza sativa*	<10	
*Ipomoea batatas*	467.30–523.90	
*Abelmoschus esculentus*	56.30–317.20	
*Glycine max*	33.40–42.50	
*Pisum sativum*	244.70–294.00	[16]
*Lens culinaris*	168.60–289.10	
*Vicia faba*	241.50–291.40	
*Cicer arietinum*	92.20–214.00	
Black Tea	78.00–112.00	[17]
Green Tea	40.00–50.00	
Red wine and beer	<1	
Carrot juice	5.81–6.20	
Tomato juice	1.43–4.43	
Milk	<10	

## 2. Oxalate Utilization by Flora Intestinal Bacteria

The use of oxalate by anaerobic and facultative anaerobic intestinal bacteria is an important process that plays a key role in the gut microbiome [18]. The ability of these bacteria to utilize oxalate contributes to maintaining gut health and metabolism by regulating the concentration of this molecule and preventing potential health-damaging effects. The human body harbors a diverse and constantly evolving community consisting of hundreds of different types of microorganisms, with the majority residing in the digestive system [19]. The composition of the microbiota in the intestines of adults is unique to each person. It tends to remain relatively consistent, although it may undergo fluctuations in response to dietary changes or the consumption of antibiotics. These variations can occur over time within an individual, highlighting the complex and dynamic nature of the interactions between the host and its gut microbiome [20].

Intestinal bacteria are responsible for carrying out a variety of biochemical reactions that have the potential to impact human health and nutrition significantly. Certain bacterial genera, such as *Lactobacillus* and *Bifidobacterium*, have gained extensive importance as probiotics in both food products and pharmaceuticals due to their natural occurrence and beneficial contributions to human health. These probiotics are known for their positive effects on the human body [21,22,23]. Intestinal bacteria play a crucial role in breaking down various dietary substances that are indigestible by humans, including oxalate, which is a compound that can be degraded by these bacteria. Oxalic acid, a simple dicarboxylic acid, is a substance that can be harmful in large quantities and is generally not the main source of energy for most bacteria because it yields a low amount of energy during the metabolic process. Despite its potential toxicity, oxalic acid can be metabolized by intestinal bacteria, showcasing the complex interactions between these microorganisms and the substances present in the human digestive system [24]. There are two main categories of oxalotrophs known as the “generalist oxalotrophs”, which exhibit versatility in utilizing various substrates for fermentation apart from oxalate, and the “specialist oxalotrophs”, which rely predominantly on oxalate as their primary carbon and energy source. These distinct classifications reflect the diverse metabolic capabilities and adaptations of microorganisms in response to their environmental niches [25].

### 2.1. Oxalobacter formigenes

*Oxalobacter formigenes* is a Gram-negative, obligatory anaerobic, rod or curve-shaped, non-motile, and non-spore-forming bacterium, and it has been classified within the following taxonomic groups: Bacteria (domain); Proteobacteria (phylum); Betaproteobacteria (class); Burkholderiales (order); *Oxalobacteraceae* (family); *Oxalobacter* (genus); *formigenes* (species). This bacterium plays a crucial role in various biological processes and ecological systems [26]. The optimal growth conditions for *O. formigenes* in culture are achieved in anaerobic settings with a pH ranging from 6 to 7, utilizing a CO_2_-bicarbonate buffered undefined medium that includes minerals, oxalate, acetate, and a small portion of yeast extract. Although yeast extract is not mandatory, it has been observed to enhance the growth of certain strains of *O. formigenes*, particularly during the initial stages of isolation from the gastrointestinal tract. Irrespective of the presence or absence of yeast extract, acetate’s minimal concentration (ranging from 0.5 to 2 mM) is incorporated since *O. formigenes* utilizes small quantities of carbon from acetate, alongside carbon dioxide, to synthesize cellular biomass. It is important to note that acetate by itself is incapable of sustaining the growth of this microorganism; indeed, no more than 60 different compounds that have been experimented with to date have exhibited growth-promoting properties for *O. formigenes* [26]. Acetate is crucial for cell synthesis and is essential for some, if not all, strains.

The breakdown of oxalate leads to medium alkalization, where formate is generated in approximately equal amounts to the oxalate metabolized. Isolates of these strains have been recovered from diverse sources such as cattle and sheep rumens and cecal and fecal samples from various animals, including humans, guinea pigs, swine, and domestic and wild rats. Moreover, they have been found in freshwater lakes and marine sediments. These bacteria likely inhabit numerous other anaerobic environments [27]. *O. formigenes* can colonize the gastrointestinal tract and diminish the oxalate concentration in the urine after an oxalate challenge, thereby mitigating the probability of developing calcium oxalate kidney stones (Table 2). Current research indicates that anaerobic bacteria present in the colon, such as *O. formigenes*, can metabolize harmful substances within the intestinal environment. This highlights the pivotal role of *O. formigenes* in modulating oxalate levels and potentially preventing the formation of calcium oxalate kidney stones through its unique biochemical mechanisms [28].

### 2.2. Lactic Acid Bacteria and Bifidobacteria

Lactic acid bacteria, which are classified as Gram-positive bacteria, are characterized by their inability to produce spores except for the genus *Sporolactobacillus*, thus setting them apart from other bacterial groups. While some LAB thrive in aerobic environments, others prefer anaerobic conditions; the majority of species exhibit the ability to adapt to both aerobic and anaerobic conditions, classifying them as facultative anaerobes. Through the process of fermentation, these bacteria effectively convert carbohydrates into lactic acid, which is the major fermentation product. This metabolic pathway not only yields lactic acid but also results in the formation of various organic acids and other metabolites that significantly influence the overall sensory profile of the end product, contributing to its flavor, texture, and aroma, thereby enhancing its desirability [38,39].

The groundwork for the systematic classification of LAB was established in 1919, marking a pivotal moment in the understanding of these microorganisms. It is a type of classification framework revolving around specific criteria, including the assessment of glucose fermentation capabilities, cell morphology, the capacity to utilize sugars as carbon sources, and growth patterns under diverse temperature conditions. Initially, the classification encompassed four distinct genera within this bacterial group: *Lactobacillus*, *Pediococcus*, *Leuconostoc*, and *Streptococcus*. However, with the rapid advancements in genetic methodologies, numerous additional genera have been incorporated into this taxonomic group, broadening the spectrum of lactic acid bacteria. Among the newly identified genera are *Aerococcus*, *Alloiococcus*, *Carnobacterium*, *Dolosigranulum*, *Enterococcus*, *Lactosphaera*, Melissococcus, *Oenococcus*, *Sporolactobacillus*, *Tetragenococcus*, *Vagococcus*, and *Weissella*, reflecting the expanding diversity and complexity within this bacterial family [40,41].

In 2020, the taxonomic classification of the genus *Lactobacillus* underwent a significant reorganization due to the remarkable progress in genetic engineering and genetic diagnosis technologies. This reorganization divided the genus *Lactobacillus* into 25 genera, each representing a distinct cluster of bacterial species. These newly classified genera include *Acetilactobacillus*, *Agrilactobacillus*, *Amylolactobacillus*, *Apilactobacillus*, *Bombilactobacillus*, *Companilactobacillus*, *Dellaglioa*, *Fructilactobacillus*, *Furfurilactobacillus*, *Holzapfelia*, *Lacticaseibacillus*, *Latilactobacillus*, *Lactiplantibacillus*, *Lapidilactobacillus*, *Lentilactobacillus*, *Levilactobacillus*, *Ligilactobacillus*, *Limosilactobacillus*, *Liquorilactobacillus*, *Loigolactobacilus*, *Paralactobacillus*, *Paucilactobacillus*, *Schleiferilactobacillus*, and *Secundilactobacillus*, as detailed by Zheng et al. [42]. This taxonomic revision reflects the increasing precision and depth of our understanding regarding bacterial diversity and evolution, highlighting the intricate interplay between technological advancements and biological classification systems in the field of microbiology.

*Bifidobacterium* was first identified in 1924. Gram-positive bacteria that do not retain acid-fast stains, do not form spores, and are non-motile. Typically, cells exhibit irregular staining patterns when subjected to methylene blue. These organisms thrive in anaerobic environments; however, certain species show a tolerance towards oxygen only in the presence of carbon dioxide. Recently identified species like *B. psychraerophilum*, *B. scardovii*, and *B. tsurumiense* have demonstrated the ability to flourish under aerobic conditions. The most favorable temperature range for growth is reported to be between 37 and 41 °C, except *B. mongoliense*, which shows optimal growth at 30 °C. The lowest temperature suitable for growth ranges from 25 to 28 °C, except for *B. mongoliense* and *B. psychraerophilum*, which can thrive at 15 °C and 8 °C, respectively. On the other hand, the maximum temperature conducive to growth spans from 43 to 45 °C, except for *B. thermacidophilum*, which can thrive up to 49.5 °C. Notably, the ability to grow at 45 °C is a distinguishing factor between strains isolated from animals and those from humans, as most animal strains can survive at this temperature while human strains cannot. The ideal pH for initial growth falls within the range of 6.5 to 7.0; these bacteria do not increase at pH levels ranging from 4.5 to 5.0, except *B. thermacidophilum*, which can still grow at pH 4.5 or pH 8.0 to 8.5 [43].

Several recent research studies have provided substantial evidence regarding the capacity of lactic acid bacteria, specifically the genera *Bifidobacterium* and *Lactobacillus*, to degrade oxalates and transform them into formate and CO_2_ as highlighted in the works of Murru et al. [44] and Chamberlain et al. [45]. In the study conducted by Sadaf et al. [46], it was mentioned that they isolated four distinct types of *Lactobacillus* bacteria, which are *Lactobacillus acidophilus*, *Lactiplantibacillus plantarum*, *Lacticaseibacillus casei*, and *Lacticaseibacillus zeae*, and thoroughly investigated their oxalate breakdown capacity. The research findings revealed that *Lactobacillus acidophilus* exhibited a superior efficiency in oxalate degradation compared to the other bacterial isolates. Additionally, the research by Karamad et al. [47] reported that *Lactobacillus acidophilus* bacteria demonstrated the ability to break down oxalates present in artificially prepared gastric fluid by an impressive 48% efficiency rate. These studies collectively shed light on the promising potential of lactic acid bacteria, particularly *Lactobacillus* strains, in effectively metabolizing oxalates into more benign byproducts, which could have significant implications in various fields such as nutrition, medicine, and biotechnology (Table 3). Furthermore, the research outcomes underscore the importance of exploring the diverse capabilities of probiotic bacteria in promoting gastrointestinal health and potentially mitigating the risks associated with oxalate-related disorders. The findings presented in these studies contribute valuable insights to the existing body of knowledge on the metabolic activities of lactic acid bacteria and their role in oxalate degradation mechanisms. Overall, the research outcomes suggest a promising avenue for future investigations focusing on harnessing the oxalate-degrading potential of lactic acid bacteria for therapeutic or dietary interventions aimed at improving human health and well-being.

### 2.3. Other Gut Bacteria

Many bacterial species inhabit the small intestine of the host (human or animal). They are natural flora and have the ability to decompose oxalate and reduce the concentration of oxalate in the blood and urine. Anaerobic conditions were used to isolate an oxalate-erode degrading *Enterococcus faecalis* bacterium from human feces. For the bacteria to continue reducing oxalate, they needed a low nutritional environment and frequent sub-culturing. SDS-PAGE analysis revealed that *E. faecalis* synthesized three proteins (65, 48, and 40 kDa) that were not formed by the non-oxalate-degrading *E. faecalis* [56]. Another investigation discovered a single *Providencia rettgeri* bacterial isolate that displayed two proteins (65 kDa and 48 kDa) on SDS-polyacrylamide gel electrophoresis that were absent in *P. rettgeri* that did not break down oxalate. In Western blotting, antibodies interacted with the *P. rettgeri* strain’s 65 and 48 kDa proteins. An *Oxalobacter* formyl-coenzyme from *O. formigenes* under highly stringent circumstances, a transferase gene probe interacted with *P. rettgeri* chromosomal DNA on Southern blotting. In contrast, an *Oxalobacter formigenes* oxalyl-coenzyme with identical circumstances did not cause a decarboxylase gene probe to respond. Oxalate decarboxylase (OXC), a manganese-dependent enzyme, is involved in the catalysis of oxalate oxidation to carbon dioxide with the formation of hydrogen peroxide. The ability of this OXC enzyme to purify from the *Pseudomonas* strain confirmed that it can be used for the diagnosis of oxalate-related disorders through microtiter plate analysis [57]. A previous study found that *Pseudomonas* sp. OXDC12 could produce and purify OXC up to 45.3 times, with an overall yield of 7%. The pure OXC may be a hexameric enzyme because it only showed one band, measuring around 40 kDa on SDS-PAGE and 240 kDa on Native PAGE [58].

### 2.4. Mechanisms of Bacterial Oxalate Degradation

Oxalate is a compound produced by many edible plants. As a terminal metabolite in the liver of mammals, it acts as a toxin that has detrimental effects on human health [59]. Endogenous oxalate-degrading enzymes are absent in humans and other mammals, leading them to depend on the gut microbiota for this crucial metabolic process. Several types of bacteria within the gastrointestinal tract have been recognized for their ability to break down oxalate through various in vitro as well as in vivo studies, with notable examples including *Oxalobacter formigenes*, various species of *Lactobacillus*, different *Bifidobacterium* strains, and members of the *Enterobacteriaceae* family [60].

*Oxalobacter formigenes* are particularly remarkable due to their utilization of oxalate as the exclusive source of energy, resulting in the stimulation of host oxalate secretion into the colonic lumen. This bacterium, in conjunction with other microorganisms, harbors crucial oxalate-degrading enzymes (ODEs) such as oxalyl-CoA decarboxylase (OXC) and formyl-CoA transferase (FRC), which play a vital role in oxalate metabolism. These pivotal enzymes are governed by genes that are commonly present in the genetic makeup of oxalate-degrading bacteria. One of the interesting facts is that taxa encoding FRC and OXC are exclusively of bacteria only, whereas those encoding OXDD can be of both fungal and bacterial origin. Through experimental investigations, it has been demonstrated that the colonization of mice with *O. formigenes* leads to a substantial reduction in urinary oxalate levels, thereby confirming its significance in the degradation of oxalate. Analogously, various other oxalate-degrading bacteria have exhibited the ability to diminish urinary oxalate levels in rodent models of hyperoxaluria, underscoring their potential effectiveness in regulating oxalate concentrations within living organisms [60,61].

In the microbiota of a healthy human, the *oxc* gene is commonly transcribed by various species, including *E. coli*, *O. formigenes*, and members of Muribaculaceae, *Bifidobacterium*, and *Lactobacillus*, with *E. coli* and *O. formigenes* being the most predominant among them. However, under conditions of diseases such as Inflammatory Bowel Disease (IBD), the transcription of these genes may undergo a significant reduction. It has been observed that despite the presence of *frc* and *oxc* genes in patients with IBD, the actual expression levels of these genes are considerably lower in comparison to those in healthy individuals, which is associated with the depletion of *O. formigenes*, a key oxalate-degrading bacterium. The intricate relationship between the transcription of these genes and the microbial composition in the gut highlights the potential implications of dysbiosis in disease pathogenesis, particularly in the context of IBD [32,62].

**Figure 2:**
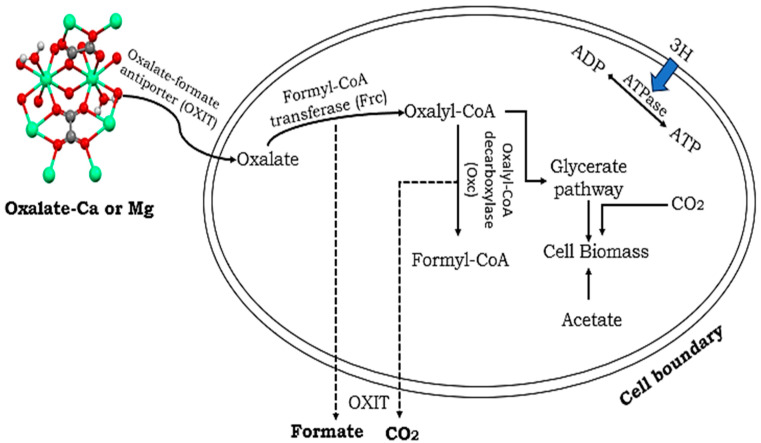
Decomposition of oxalate molecules through biological processes by different species of bacteria within the host.

The differential transcription observed signifies a significant shift in the functional dynamics of the microbiota, indicating that the abundance of genes may not always align with actual functional activity. Particularly, the transcriptional activity of *O. formigenes* emerges as a central player in the overall oxalate degradation pathway (ODP), underscoring its crucial role as an oxalate autotroph and the prevalence of FRC and OXC proteins across various growth phases (Figure 2). These findings underscore the paramount importance of relying on transcriptional evidence rather than mere gene abundance when assessing microbiome functions associated with oxalate breakdown. It is crucial to acknowledge that environmental variables, such as dietary constituents and pH levels, have a significant impact on the effectiveness of oxalate-degrading bacteria. The expression levels of oxc and *frc* genes, which directly influence oxalate degradation efficiency, are notably influenced by these external factors, highlighting the intricate nature of oxalate metabolism within diverse environmental frameworks [59,60].

## 3. Health Implications

The gut microbiome, which consists of many micro-organisms like bacteria, viruses, fungi, and archaea, is fundamentally important in maintaining human health through its influence on processes such as digestion, absorption of nutrients, and regulation of the immune system. The disruption or dysbiosis of the gut microbiome, characterized by an imbalance in its composition, has been associated with the development and progression of various medical conditions, including inflammatory bowel disease (IBD), diabetes, and kidney stone disease. These findings underscore the intricate relationship between the gut microbiome and human health, highlighting the potential therapeutic implications of targeting the microbiome to manage and prevent a wide range of diseases.

### 3.1. Inflammatory Bowel Disease (IBD)

The gut microbiome composition in patients suffering from inflammatory bowel disease (IBD) shows a significant deviation from that of individuals in good health, characterized by a decrease in bacterial diversity and a decline in beneficial species such as butyrate-producing bacteria while being marked by an increase in opportunistic species referred to as pathobionts [63]. Metabolites generated by the gut microbiota, including short-chain fatty acids, tryptophan metabolites, and bile acids, have been linked to the onset and advancement of IBD, emphasizing the critical role of these compounds in the disease process. The alterations in the gut microbiome and its metabolites highlight the intricate interplay between the microbial community residing in the gut and the pathophysiology of IBD, shedding light on potential therapeutic targets for managing this complex disorder. Alterations in the gut microbiota have been correlated with two prevalent gastrointestinal disorders, namely inflammatory bowel disease (IBD) and irritable bowel syndrome (IBS). In this study, researchers conducted a case-control examination utilizing shotgun metagenomic sequencing of fecal samples from 1792 individuals diagnosed with IBD and IBS in comparison to control subjects from the general population [64]. The findings from the examination of the gut microbiome composition in Saudi individuals diagnosed with inflammatory bowel disease (IBD) revealed the presence of three detrimental bacterial biomarkers: the Paraprevotellaceae, the Muribaculaceae families belonging to the Bacteroidetes phylum, and the Leuconostocaceae family from the Firmicutes phylum, which exhibited a higher relative prevalence in healthy subjects as opposed to those suffering from IBD. Moreover, it was observed that specific microbiota signatures at particular genera and species levels, such as *Prevotella copri*, *Bifidobacterium adolescentis*, *Ruminococcus callidus*, *Coprococcus* sp., *Ruminococcus gnavus*, *Dorea formicigenerans*, *Leuconostoc*, *Dialister*, *Catenibacterium*, *Eubacterium biforme*, and *Lactobacillus mucosae*, were virtually absent in nearly all IBD patients. In contrast, *Veillonella dispar* was completely absent among all healthy individuals [65].

### 3.2. Kidney Stone Disease

The research carried out by Pisani et al. [63] has demonstrated a connection between the gut microbiome in patients with inflammatory bowel disease (IBD) and increased levels of fecal oxalate, especially in individuals diagnosed with Crohn’s disease (CD) and ulcerative colitis (UC). This association has the potential to raise the likelihood of developing kidney stones. Notably, the increased fecal oxalate concentrations in patients with CD are primarily observed in those with ileocolonic inflammation, indicating that the specific location of IBD within the gastrointestinal tract plays a critical role in assessing the associated risks [63,64].

Kidney stone disease, which is a condition that impacts an estimated 10 to 15% of the populace on a global scale, exhibits a significant correlation with the phenomenon of dysbiosis within the gut microbiota [66]. The predominant classification of kidney stones, specifically calcium oxalate stones, constitutes a substantial 76% of all documented cases of this medical condition [67]. The state of dysbiosis observed in patients afflicted with kidney stones is distinguished by a particular microbial profile within the gut, which may have the potential to significantly affect both the formation of these stones and their subsequent recurrence [68]. The persistence of kidney stones over an extended period can lead to a multitude of severe health complications, which may include but are not limited to urinary tract obstruction, the development of infections, and the potential for irreversible damage to renal structures. Innovative and individualized therapeutic approaches, including but not limited to microbial supplementation, the use of probiotics, the implementation of synbiotics, and dietary modifications informed by the unique characteristics of the gut microbiome, have demonstrated considerable promise in the realms of both the prevention of stone formation as well as the reduction of recurrence rates [69].

## 4. Future Perspectives and Directions

The significance of the gut microbiome in the manifestation and progression of various pathological conditions underscores the vast potential for the development and implementation of personalized medical interventions that are tailored to individual patient profiles. A comprehensive understanding of the complex and multifaceted interactions that exist between dietary practices, the diverse composition of the microbiota, and the inflammatory responses that occur within the body could ultimately facilitate the formulation of innovative strategies aimed at both the prevention and the treatment of diseases such as Inflammatory Bowel Disease (IBD) and Kidney stones (KSs). The identification and validation of promising biomarkers found within the blood and fecal specimens may prove instrumental in enhancing the accuracy of diagnoses as well as in forecasting the trajectory of disease progression and the efficacy of therapeutic interventions, thereby paving the way for the advent of more customized and effective treatment regimens that are specifically designed to meet the unique needs of each patient.

The persistent quest for innovative microbial strains exhibiting superior fermentative capabilities and probiotic characteristics is anticipated to play a crucial role in forthcoming developments. Scholars advocate for the employment of bacteriocin-producing strains as initial inoculate to effectively combat foodborne pathogens. Nonetheless, the possible adverse effects associated with bacteriocins, particularly their toxicity, require thorough examination. Although evidence suggests that bacteriocins are typically non-immunogenic and display low cytotoxicity, there have been instances documenting considerable cytotoxic repercussions. These effects may fluctuate according to concentration and the type of target cells, thus warranting additional research.

The prospective trajectory of microbiome research is poised to advance significantly through the meticulous amalgamation of metabolomic data with an array of other critical biomarkers, which encompass the proteome as well as circulating antibodies, all of which should be examined across a wide spectrum of diverse and multiethnic cohorts that reflect the complexity of human health. Such a comprehensive and integrative methodological framework has the potential not only to substantially enhance the precision and reliability of diagnostic tests but also to yield profound insights into the intricate nature of disease heterogeneity and its progression over time, thereby informing targeted therapeutic strategies. Forthcoming research endeavors must concentrate on mechanistic inquiries to substantiate the significance of novel metabolites or pathways linked to ailments such as inflammatory bowel disease (IBD) and to prioritize biomolecules for the formulation of innovative therapeutic strategies. Such inquiries will be essential in differentiating metabolites that instigate diseases from the by-products resulting from inflammatory processes. Previous prospective cohort studies have already delineated serum metabolomic markers, including sarcosine, carnitine, propionyl-l-carnitine, and sorbitol, which correlate with clinical relapses in individuals diagnosed with IBD.

Ultimately, forthcoming investigations seek to reassess various methodologies employed in research centered on advantageous microorganisms and bacteriocins as preservative agents. This entails the identification of novel bacterial strains sourced from fermented food products, the examination of bacteriocins produced by these microorganisms, and the evaluation of their viability as probiotics. Scholars advocate for empirical studies to incorporate bacteriocins into particular food items and to analyze their effectiveness in mitigating foodborne pathogens. This domain presents considerable promise for the creation of pioneering strategies aimed at managing oxalate degradation while simultaneously improving food safety and overall quality.

## 5. Conclusions

It is possible to conclude that the intricate ecologies of various bacterial strains serve a significantly crucial function in enhancing their overall efficiency in the solubilization processes of mineral compounds, which are essential for numerous biological and geological functions. Various strains belonging to the genera *Oxalobacter*, *Lactobacillus*, and *Bifidobacterium* have demonstrated considerable potential as effective probiotic supplements, which can play a pivotal role in meticulously regulating and monitoring the levels of rock-forming salts that accumulate within the kidneys, thereby contributing to the maintenance of renal health.

## Figures and Tables

**Table 2:** An overview of earlier research on the application of *Oxalobacter formigenes* in urolithiasis prophylaxis.

Bacteria Strains	Study Type	Study Condition	Study Outcome	References
*O. formigenes* DSM 4420	In vitro	Growth on 1.35 (g/L) inulin, 36.56 (g/L) glucose, 26 (mmol/L) ammonium oxalate, pH = 6	The achievement of reducing the levels of oxalate has resulted in reaching 61.74%	[29]
*O. formigenes* DSM 4420	In vitro	Growth on SM medium was composed of 5 mL of filtered sterilized 20 mmol/L ammonium oxalate and 40 g/L dextrose) added to 5 mL of base medium	The achievement of reducing the levels of oxalate has resulted in reaching 98.22%	[30]
*O. formigenes* DSM 4420	In vitro	Growth on SM medium was composed of 5 mL of filtered sterilized 20 mmol/L ammonium oxalate and 40 g/L dextrose) added to 5 mL of base medium	The achievement of reducing the levels of oxalate has resulted in reaching 98.00%	[31]
*O. formigenes* strain OxWR	In vivo	Male rats were segregated into 6 subcategories: the first subgroup received a standard diet; the second subgroup was provided with a diet rich in oxalate; subgroups three to six were administered an oxalate-rich diet along with an esophageal gavage of 10^3^, 10^5^, 10^7^, and 10^9^ CFU/g *O. formigenes*, respectively, with each feeding lasting for two weeks. Samples of urine were also gathered to evaluate the extent of oxalate extraction.	The group that received an oxalate-rich diet and *O. formigenes* exhibited decreased levels of urine oxalate compared to the group that solely received an oxalate-rich diet.	[32]
*Oxalobacter* sp.	In vivo	Male rats were segregated into two distinct cohorts. The first cohort underwent colonization through esophageal gavage with a 1.5 mL inoculum containing 2 × 10^9^ CFU derived from a 24 h culture of a wild rat strain of *O. formigenes*, whereas the second cohort remained noncolonized. The diet administered to both cohorts of rats was consistent. Furthermore, urinary samples were collected to evaluate the extent of oxalate extraction.	Rats colonized by *Oxalobacter* sp. exhibited a reduction in urinary oxalate levels compared to non-colonized rats fed the same diet.	[33]
*O. formigenes*	In vivo	A total of 247 stool samples from patients were obtained to evaluate the existence of *O. formigenes*. Additionally, samples of urine were obtained to evaluate the extent of oxalate extraction.	The frequency of *O. formigenes* was found to be 17% in individuals diagnosed with the condition, while among the control group, it was 38%. Additionally, harboring *O. formigenes* was linked to a 70% decrease in the likelihood of experiencing repeated occurrences of calcium oxalate stones.	[34]
*O. formigenes*	In vivo	A total of 37 individuals who form calcium oxalate stones, 26 were found to have a negative test result for *O. formigenes* and were then analyzed in comparison to the remaining 11 patients who tested positive. These patients were requested to provide 24 h urine samples while following both a diet of their own choice and a standardized diet.	According to the results, *O. formigenes* reduces the amount of oxalate in the intestinal solution that is accessible for absorption at steady rates, which lowers the amount of oxalate excreted in the urine.	[35]
*O. formigenes*	In vivo	The research was segmented into two distinct dietary phases, each lasting for a three-week duration. During the initial phase, the participants’ intake of dietary oxalate was manipulated, with a daily consumption ranging from 50 mg in the first week to 250 mg in the second week and 750 mg in the third week. Subsequently, in the second phase of the study, the focus shifted towards varying dietary calcium intake levels, with participants consuming 400 mg daily in the first week, 1000 mg in the second week, and 2000 mg in the third week. Following the completion of these dietary interventions, samples of urine and stool were collected from the participants to be analyzed for stone risk parameters and the levels of *O. formigenes*, a key bacterium involved in oxalate metabolism in the gut. The utilization of these samples allowed for the assessment of potential correlations between dietary oxalate and calcium intake levels and their impact on stone formation risk factors and *O. formigenes* abundance in the participants’ microbiota.	Levels of *O. formigenes* significantly rose by a factor of 10 as the dietary oxalate intake surged by 15 times. However, a decline in the *O. formigenes* content was observed with the escalation in calcium consumption.	[36]
*O. formigenes* ATCC 35274	In vivo	*O. formigenes* has been observed to engage with the colonic epithelium, resulting in the stimulation of colonic oxalate secretion, consequently leading to a reduction in urinary oxalate excretion. In this research article, the influence of *O. formigenes* culture conditioned medium on the uptake of apical 14C-oxalate by human intestinal Caco-2-BBE cells was examined. The findings revealed significant differences in apical 14C-oxalate uptake when compared to the control medium, suggesting a potential role of *O. formigenes* in modulating oxalate transport mechanisms in the intestinal cells.	*O. formigenes*-derived bioactive factors stimulate oxalate transport in intestinal cells through mechanisms. The reduction in urinary oxalate excretion in hyperoxaluric mice treated with *O. formigenes* conditioned medium reflects the in-via retention of biological activity and the therapeutic potential of these factors.	[37]

**Table 3:** A summary of outcomes obtained from previous studies on the application of lactic acid bacteria and bifidobacteria in kidney stone disease.

Bacteria Strains	Study Type	Study Condition	Study Outcome	References
*Lactiplantibacillus plantarum*	in vivo	In this work, researchers used a host lactate dehydrogenase promoter to create a recombinant strain of *Lb. plantarum* that constitutively over-expressed *B. subtilis* oxalate decarboxylase.	The recombinant *Lb. plantarum* was able to break down almost 90% of the oxalate. Furthermore, the recombinant strain exhibited a greater tolerance to oxalate up to 500 mmol/L.	[48]
*Lactobacillus acidophilus* ATCC 4356,	In vivo	Utilizing *Lb. acidophilus*, oxalate degradation activity was measured where a Box–Behnken method was used with four major variables: pH, glucose, sodium oxalate, inulin, ammonium, yeast extract, sodium acetate, inoculum age, and size.	Under optimized conditions of major variables, including pH, glucose, inoculum age & size, the breakdown of oxalate attained 48.94% of its starting concentration. Additionally, the breakdown of oxalate in tea (the most popular hot beverage in many nations) was studied at different temperatures, times of day, and concentrations of glucose. Under ideal circumstances, oxalate content increased from 264 to 24 mg/100 mL, or 98.86%.	[47]
*Lactobacillus*	Clinical study (200 patients)	Serum, urine, and feces samples were used in this case-control investigation. There were two equal groups of 200 participants in total that made up the research population. They were chosen from among urologists’ patients who had urinary tract stones, regular folks, and visiting patients. The evaluation included the amount of calcium, oxalate, and citrate in the urine samples, parathyroid and calcium in the blood samples, and the degrading activity of all the participants’ fecal *Lactobacillus* strains.	The findings showed that compared to normal individuals, the patients had greater blood levels of parathyroid hormone and higher levels of oxalate and calcium in their urine. On the other hand, normal individuals had greater urine levels of citrate. The ability of *Lactobacillus* isolated from the patients to degrade oxalate differed significantly from that of their normal counterparts.	[49]
*Lactobacillus acidophilus* and *Lb. gasseri*	in vitro	Using ultra-high-performance liquid chromatography-high resolution mass spectrometry, authors compared and characterized the metabolomes and lipidomes of the oxalate-degrading *Lb. acidophilus* and *Lb. gasseri.*	Many species-specific differences in the metabolic profiles of these bacteria were observed. Further, using ^14^C liquid scintillation counting, the oxalate-degrading capacity of *Lb. acidophilus* and *Lb. gasseri* in vitro, even in the presence of other preferred carbon sources, was also validated.	[45]
*Lactobacillus paragasseri* UBLG-36	in vitro	*Lb. paragasseri* UBLG-36 had genes encoding for oxalate catabolic enzymes, including oxalyl coenzyme A decarboxylase (*oxc*) and formyl coenzyme A transferase (*frc*). The in vitro oxalate degradation experiment demonstrated the great efficacy of UBLG-36 in oxalate breakdown.	*Lb. paragasseri* had a previously unreported oxalate degradation rate of 45%. Since *Lb. paragasseri* is a relatively new bacterium; there are not many genomic papers on it. The genome sequence study is the first to highlight and explain this species’ probiotic potential as well as its capacity to break down oxalate.	[50]
*Lacticaseibacillus casei*, *Limosilactobacillus fermentum*, *Lb. acidophilus* and *Lb. gasseri*	in vitro	Twenty-four of the 62 oxalate-degrading bacteria showed a zone larger than 10 mm, according to early characteristics. It was determined that lactic acid bacteria were the most powerful bacterial strains.	At a concentration of 50 µg of calcium oxalate, *Lb. acidophilus* was able to degrade the material to 75.6 ± 0.865%. A date waste-derived lactic acid bacterium shown to encourage anti-urolithiasis action.	[51]
*Streptococcus thermophilus*, *Limosilactobacillus fermentum* NRAMJ5, and *Lb. gastricus* NRAMJ5	in vitro and in vivo	In this study, rats given a diet rich in oxalate were used as test subjects, and the effects of two probiotic preparations were seen on urine functions. Five groups, each including eight rats, were formed from a total of forty male Sprague-Dawley rats. There were two sets of participants: one was utilized as a high-oxalate positive control group and was fed a regular, balanced diet; the other was used as a negative control group. A diet high in oxalate was also given to the remaining three groups, along with oral dosages of the probiotic preparations.	Different measurements, including urine pH, aspartate transaminase (AST) activity, urea, uric acid, phosphorus, calcium, magnesium, and creatinine, were measured from blood and urine samples from rats. The study’s conclusions demonstrated the beneficial effects of these probiotic formulations on kidney function, perhaps through increased intestinal oxalate metabolism and/or decreased gastrointestinal tract oxalate absorption. Furthermore, both preparations resulted in a rise in the counts of lactobacilli numbers in the fecal samples, whereas coliform counts dropped in comparison to the baseline.	[52]
*Bifidobacterium* *adolescentis* ATCC 15703, *B. animalis* subsp. *lactis* DSM 10140, BA30, Bb12, BI07, and L15, *B. bifidum* S16, *B. breve* ATCC 15700 and BBSF, *B. catenulatum* B665, *B. longum* biotype *longum* S123, ATCC 15707, and W11, and *B. longum* ATCC 27533	in vitro	The enzymatic breakdown of oxalate by 14 *Bifidobacterium* strains, grown in media rich in oxalate, was quantified using capillary electrophoresis. The detection of the *oxc* gene was achieved through PCR.	Out of 14, the oxalate-degrading activity was observed only in the five *B. animalis* subsp. *lactis* strains (DSM 10140, BA30, Bb12, BI07, and L15), resulting in complete consumption of oxalate after 5 days of incubation. In contrast, none of the other tested *Bifidobacterium* strains displayed any oxalate degrading activity.	[53]
*Bifidobacterium animalis* subsp. *lactis* DSM 10140 and *B. adolescentis* ATCC 15703	in vivo	Two strains of *Bifidobacterium* were administered to both wild-type mice and mice lacking the hepatic enzyme alanine-glyoxylate aminotransferase while being fed a diet supplemented with oxalate. Subsequently, urine samples were obtained to evaluate oxalate extraction levels, and fecal samples were collected to monitor the colonization status of mice with *Bifidobacterium* through weekly PCR analysis.	The introduction of *B. animalis* subsp. *lactis* resulted in a notable reduction in the excretion of urinary oxalate in both wild-type and Agxt−/− mice when compared to the use of *B. adolescentis*. The wild-type mice exhibited a greater degree of colonization by *B. animalis* subsp. *lactis* in comparison to Agxt−/− mice.	[54]
*Lacticaseibacillus casei* and *Bifidobacterium breve*	Clinical study 14 patients	For four weeks, the patients were given an oxalate-supplemented diet; in the final two weeks, they were given a lyophilized mixture of *Lacticaseibacillus casei* and *Bifidobacterium breve* after meals. Lastly, urine samples were taken to evaluate the extraction amount of oxalate.	Compared to pre-treatment, seven out of fourteen patients showed a decrease in oxaluria following probiotic preparation; in four cases, this reduction was greater than 25%, and in two cases, it was as high as 50%.	[55]

## Data Availability

Data is contained within the article.
